# 2-Chloro­ethyl 2-(5-bromo-3-methyl­sulfinyl-1-benzofuran-2-yl)acetate

**DOI:** 10.1107/S1600536809012847

**Published:** 2009-04-10

**Authors:** Hong Dae Choi, Pil Ja Seo, Byeng Wha Son, Uk Lee

**Affiliations:** a Department of Chemistry, Dongeui University, San 24 Kaya-dong Busanjin-gu, Busan 614-714, Republic of Korea; b Department of Chemistry Pukyong National University 599-1 Daeyeon 3-dong, Nam-gu, Busan 608-737, Republic of Korea

## Abstract

In the title compound, C_13_H_12_BrClO_4_S, the O atom and the methyl group of the methyl­sulfinyl substituent lie on opposite sides of the plane of the benzofuran fragment. There is a mean deviation of 0.016 (4) Å from the least-squares plane defined by the nine constituent benzofuran atoms. The crystal structure is stabilized by aromatic π–π inter­actions between the benzene rings of neighbouring mol­ecules [centroid–centroid distance = 3.689 (7) Å]and by a weak C—H⋯π interaction between an H atom of the methylene group bonded to the carboxylate O atom and the benzene ring of an adjacent molecule. In addition, the crystal structure exhibits weak non-classical inter­molecular C—H⋯O hydrogen bonds. The chloro­ethyl group is disordered over two positions, with refined site-occupancy factors of 0.767 (6) and 0.233 (6).

## Related literature

For the crystal structures of similar alkyl 2-(5-bromo-3-methyl­sulfinyl-1-benzofuran-2-yl)acetate derivatives. see: Choi *et al.* (2008*a* ▶,*b* ▶).
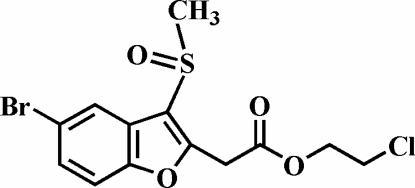


## Experimental

### 

#### Crystal data

C_13_H_12_BrClO_4_S
*M* *_r_* = 379.65
Triclinic, 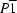

*a* = 8.495 (1) Å
*b* = 9.882 (2) Å
*c* = 10.277 (2) Å
α = 71.095 (3)°
β = 80.331 (3)°
γ = 65.012 (2)°
*V* = 739.3 (2) Å^3^
*Z* = 2
Mo *K*α radiation
μ = 3.11 mm^−1^
*T* = 298 K
0.30 × 0.20 × 0.10 mm

#### Data collection

Bruker SMART CCD diffractometer
Absorption correction: multi-scan (*SADABS*; Sheldrick, 1999 ▶) *T*_min_ = 0.475, *T*_max_ = 0.736
5479 measured reflections
2575 independent reflections
1972 reflections with *I* > 2σ(*I*)
*R*_int_ = 0.027

#### Refinement

*R*[*F* ^2^ > 2σ(*F* ^2^)] = 0.055
*wR*(*F* ^2^) = 0.133
*S* = 1.07
2575 reflections
192 parameters
57 restraints
H-atom parameters constrained
Δρ_max_ = 0.80 e Å^−3^
Δρ_min_ = −0.81 e Å^−3^

### 

Data collection: *SMART* (Bruker, 2001 ▶); cell refinement: *SAINT* (Bruker, 2001 ▶); data reduction: *SAINT*; program(s) used to solve structure: *SHELXS97* (Sheldrick, 2008 ▶); program(s) used to refine structure: *SHELXL97* (Sheldrick, 2008 ▶); molecular graphics: *ORTEP-3* (Farrugia, 1997 ▶) and *DIAMOND* (Brandenburg, 1998 ▶); software used to prepare material for publication: *SHELXL97*.

## Supplementary Material

Crystal structure: contains datablocks global, I. DOI: 10.1107/S1600536809012847/sj2602sup1.cif

Structure factors: contains datablocks I. DOI: 10.1107/S1600536809012847/sj2602Isup2.hkl

Additional supplementary materials:  crystallographic information; 3D view; checkCIF report

## Figures and Tables

**Table 1:** Hydrogen-bond geometry (Å, °)

*D*—H⋯*A*	*D*—H	H⋯*A*	*D*⋯*A*	*D*—H⋯*A*
C11*A*—H11*B*⋯*Cg*^i^	0.97	3.07	3.779 (7)	131
C3—H3⋯O4^ii^	0.93	2.60	3.483 (7)	159
C5—H5⋯O3^iii^	0.93	2.58	3.423 (7)	150
C9—H9*A*⋯O1^iv^	0.97	2.60	3.545 (6)	165
C9—H9*B*⋯O4^v^	0.97	2.34	3.294 (7)	170

Symmetry codes: (i) 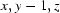; (ii) 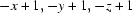; (iii) 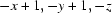; (iv) 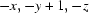; (v) 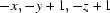. *Cg* is the centroid of the C2–C7 benzene ring.

**Fig. 1.**
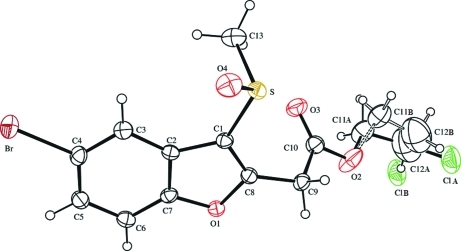
The molecular structure of the title compound with the atom numbering scheme. Displacement ellipsoids are drawn at the 50% probability level. H atoms are presented as a small spheres of arbitrary radius.

**Fig. 2.**
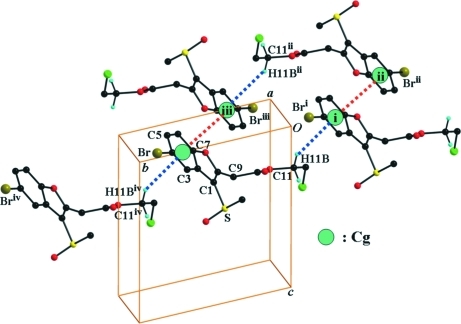
The π–π and C—H···π interactions (dotted lines) in the title compound. Cg denotes the ring centroid of the C2···C7 benzene ring. The disordered component of the chloroethyl group, part B, has been omitted for clarity as have H atoms not involved in intermolecular contacts. [Symmetry codes: (i)x, y-1, z; (ii) -x+1, -y, -z; (iii) -x+1, -y+1, -z; (iv) x, y+1, z.]

**Fig. 3.**
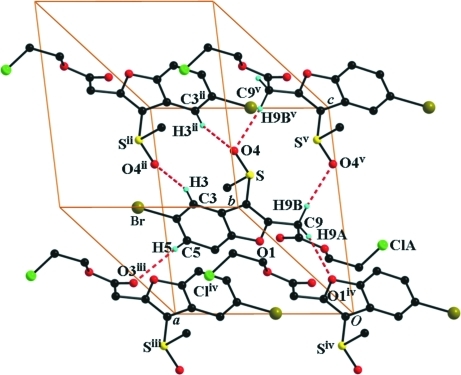
The C—H···O hydrogen bonds (dotted lines) in the title compound. The disordered component of the chloroethyl group, part B, has been omitted for clarity as have H atoms not involved in intermolecular contacts. [Symmetry codes: (ii) -x+1, -y+1, -z+1; (iii) -x+1, -y+1, -z; (iv) -x, -y+1, -z (v) -x, -y+1, -z+1.]

## supplementary crystallographic information

### Comment

This work is related to our previous communications on the synthesis and structure of alkyl 2-(5-bromo-3-methylsulfinyl-1-benzofuran-2-yl)acetate analogues, viz. isopropyl 2-(5-bromo-3-methylsulfinyl-1-benzofuran-2-yl)acetate (Choi *et al.*, 2008a) and methyl 2-(5-bromo-3-methylsulfinyl-1-benzofuran-2-yl)acetate (Choi *et al.*, 2008b). Here we report the crystal structure of the title compound, 2-chloroethyl 2-(5-bromo-3-methylsulfinyl-1-benzofuran-2-yl)acetate (Fig. 1).

The benzofuran unit is essentially planar, with a mean deviation of 0.016 (4) Å from the least-squares plane defined by the nine constituent atoms. The chloroethyl group is disordered over two positions  with site–occupancy factors of 0.767 (6) (for atoms labelled A) and 0.233 (7) (for atoms labelled B). The molecular packing (Fig. 2) is stabilized by aromatic π–π interactions between the benzene rings of neighbouring molecules, with a Cg···Cg^iii^ distance of 3.689 (7) Å (Cg is the centroid of the C2-C7 benzene ring; symmetry codes as in Fig. 2). The crystal packing is further stabilized by an intermolecular C—H···π interaction between an H atom of the methylene group bonded to the carboxylate O atom and the benzene ring of a neighbouring molecule, with a C11—H11B···Cg^i^ distance of 3.07 Å (Table 1 and Fig. 2; symmetry code as in Fig. 2, Cg^i^ is the centroid of the C2···C7 benzene ring). Additionally, the crystal structure exhibits weak, non-classical, intermolecular C—H···O hydrogen bonds; the first between a benzene H atom and the S═O unit, the second between a benzene H atom and the C═O unit, the third between an H atom of the methylene group bonded to the  carboxylate C atom and the furan O atom, and the fourth between an H atom of the methylene group bonded to the carboxylate C atom and the S═O unit, respectively (Table 1 and Fig. 3; symmetry codes as in Fig. 3).

### Experimental

77% 3-Chloroperoxybenzoic acid (271 mg, 1.1 mmol) was added in small portions to a stirred solution of 2-chloroethyl 2-(5-bromo-3-methylsulfanyl-1-benzofuran-2-yl)acetate (380 mg, 1.0 mmol) in dichloromethane (40 ml) at 273 K. After stirring for 3 h at room temperature, the mixture was washed with saturated sodium bicarbonate solution and the organic layer was separated, dried over magnesium sulfate, filtered and concentrated in vacuum. The residue was purified by column chromatography (hexane-ethyl acetate, 1:2 v/v) to afford the title compound as a colorless solid [yield 80%, m.p. 436-437 K; *R*_f_ = 0.41 (hexane-ethyl acetate, 1;2 v/v)]. Single crystals suitable for X-ray diffraction were prepared by evaporation of a solution of the title compound in chloroform at room temperature. Spectroscopic analysis: ^1^H NMR (CDCl_3_, 400 MHz) δ 3.07 (s, 3H), 3.70 (t, J = 5.48 Hz, 2H), 4.12 (s, 2H), 4.41 (t, J = 5.48 Hz, 2H), 7.41 (d, J = 8.76 Hz, 1H), 7.71 (dd, J = 8.47 Hz and J = 1.82 Hz, 1H), 8.05 (s, 1H).

### Refinement

All H atoms were positioned geometrically and refined using a riding model, with C—H = 0.93 Å for the aryl, 0.97 Å for the methylene, and 0.96 Å for the methyl H atoms.Uiso(H) = 1.2Ueq(C) for the aryl and methylene H atoms, and 1.5Ueq(C) for methyl H atoms. The chloroethyl group is disordered over two positions, with site–occupancy factors, from refinement, of 0.767 (6) (C11A—C12A—ClA) and 0.233 (7) (C11B—C12B—ClB). Both sets of C and Cl atoms were restrained using the commands ISOR (0.01), EADP, and the C—C and C—Cl distances (A & B) were restrained to 1.46 (3) and 1.55 (3) Å, respectively, using the command DFIX. Despite this the atomic displacement parameters for the C atoms of the disordered chhloroethyl group were large, reflecting additional disorder and the room temperature data collection.

### Crystal data

**Table:**

C_13_H_12_BrClO_4_S	*Z* = 2
*M**_r_* = 379.65	*F*(000) = 380
Triclinic, *P*1	*D*_x_ = 1.705 Mg m^−^^3^
Hall symbol: -p 1	Mo *K*α radiation, λ = 0.71073 Å
*a* = 8.495 (1) Å	Cell parameters from 2253 reflections
*b* = 9.882 (2) Å	θ = 2.7–25.7°
*c* = 10.277 (2) Å	µ = 3.11 mm^−^^1^
α = 71.095 (3)°	*T* = 298 K
β = 80.331 (3)°	Block, colorless
γ = 65.012 (2)°	0.30 × 0.20 × 0.10 mm
*V* = 739.3 (2) Å^3^	

### Data collection

**Table:**

Bruker SMART CCD diffractometer	2575 independent reflections
Radiation source: fine-focus sealed tube	1972 reflections with *I* > 2σ(*I*)
graphite	*R*_int_ = 0.027
Detector resolution: 10.0 pixels mm^-1^	θ_max_ = 25.0°, θ_min_ = 2.1°
φ and ω scans	*h* = −10→10
Absorption correction: multi-scan (*SADABS*; Sheldrick, 1999)	*k* = −11→11
*T*_min_ = 0.475, *T*_max_ = 0.736	*l* = −12→12
5479 measured reflections	

### Refinement

**Table:**

Refinement on *F*^2^	Primary atom site location: structure-invariant direct methods
Least-squares matrix: full	Secondary atom site location: difference Fourier map
*R*[*F*^2^ > 2σ(*F*^2^)] = 0.055	Hydrogen site location: difference Fourier map
*wR*(*F*^2^) = 0.133	H-atom parameters constrained
*S* = 1.07	*w* = 1/[σ^2^(*F*_o_^2^) + (0.054*P*)^2^ + 1.6319*P*] where *P* = (*F*_o_^2^ + 2*F*_c_^2^)/3
2575 reflections	(Δ/σ)_max_ < 0.000
192 parameters	Δρ_max_ = 0.80 e Å^−^^3^
57 restraints	Δρ_min_ = −0.80 e Å^−^^3^

### Special details

**Table:**

Geometry. All esds (except the esd in the dihedral angle between two l.s. planes) are estimated using the full covariance matrix. The cell esds are taken into account individually in the estimation of esds in distances, angles and torsion angles; correlations between esds in cell parameters are only used when they are defined by crystal symmetry. An approximate (isotropic) treatment of cell esds is used for estimating esds involving l.s. planes.
Refinement. Refinement of F^2^ against ALL reflections. The weighted R-factor wR and goodness of fit S are based on F^2^, conventional R-factors R are based on F, with F set to zero for negative F^2^. The threshold expression of F^2^ > 2sigma(F^2^) is used only for calculating R-factors(gt) etc. and is not relevant to the choice of reflections for refinement. R-factors based on F^2^ are statistically about twice as large as those based on F, and R- factors based on ALL data will be even larger.

### Fractional atomic coordinates and isotropic or equivalent isotropic displacement parameters (Å^2^)

**Table:**

	*x*	*y*	*z*	*U*_iso_*/*U*_eq_	Occ. (<1)
Br	0.68390 (9)	0.78885 (8)	0.10766 (7)	0.0571 (3)	
ClA	−0.1810 (4)	−0.0480 (3)	0.3580 (3)	0.0928 (12)	0.764 (6)
ClB	−0.0308 (13)	−0.0729 (11)	0.1579 (12)	0.0928 (12)	0.24
S	0.25778 (18)	0.40156 (16)	0.46589 (13)	0.0391 (4)	
O1	0.1648 (4)	0.5435 (4)	0.0705 (3)	0.0355 (8)	
O2	0.0404 (6)	0.1375 (5)	0.2446 (7)	0.0824 (18)	
O3	0.2843 (6)	0.1340 (5)	0.2991 (5)	0.0622 (12)	
O4	0.2572 (6)	0.5265 (5)	0.5185 (4)	0.0537 (11)	
C1	0.2533 (6)	0.4767 (6)	0.2849 (5)	0.0313 (11)	
C2	0.3429 (6)	0.5699 (5)	0.1946 (5)	0.0287 (11)	
C3	0.4664 (7)	0.6219 (6)	0.2093 (6)	0.0362 (12)	
H3	0.5126	0.5960	0.2941	0.043*	
C4	0.5167 (7)	0.7138 (6)	0.0920 (6)	0.0379 (13)	
C5	0.4497 (7)	0.7563 (6)	−0.0359 (6)	0.0395 (13)	
H5	0.4849	0.8216	−0.1110	0.047*	
C6	0.3306 (7)	0.7020 (6)	−0.0522 (6)	0.0382 (13)	
H6	0.2860	0.7271	−0.1375	0.046*	
C7	0.2815 (6)	0.6090 (6)	0.0641 (5)	0.0326 (12)	
C8	0.1505 (7)	0.4640 (6)	0.2065 (5)	0.0328 (11)	
C9	0.0354 (7)	0.3767 (6)	0.2371 (6)	0.0377 (13)	
H9A	−0.0383	0.4141	0.1598	0.045*	
H9B	−0.0392	0.3968	0.3170	0.045*	
C10	0.1380 (8)	0.2042 (7)	0.2639 (6)	0.0446 (14)	
C11A	0.1269 (13)	−0.0271 (11)	0.2556 (12)	0.069 (3)	0.764 (6)
H11A	0.2031	−0.0798	0.3329	0.083*	0.764 (6)
H11B	0.1968	−0.0422	0.1725	0.083*	0.764 (6)
C12A	−0.004 (3)	−0.092 (3)	0.275 (2)	0.162 (6)	0.764 (6)
H12A	0.0570	−0.2031	0.3143	0.195*	0.764 (6)
H12B	−0.0332	−0.0779	0.1832	0.195*	0.764 (6)
C11B	0.107 (4)	−0.044 (3)	0.343 (4)	0.069 (3)	0.24
H11C	0.2242	−0.1045	0.3153	0.083*	0.236 (6)
H11D	0.1055	−0.0480	0.4385	0.083*	0.236 (6)
C12B	−0.013 (10)	−0.105 (10)	0.324 (4)	0.162 (6)	0.24
H12C	−0.1259	−0.0542	0.3648	0.195*	0.236 (6)
H12D	0.0286	−0.2156	0.3696	0.195*	0.236 (6)
C13	0.4757 (8)	0.2580 (7)	0.4793 (7)	0.0566 (17)	
H13A	0.5553	0.3084	0.4440	0.085*	
H13B	0.4904	0.1885	0.4269	0.085*	
H13C	0.4983	0.2002	0.5740	0.085*	

### Atomic displacement parameters (Å^2^)

**Table:**

	*U*^11^	*U*^22^	*U*^33^	*U*^12^	*U*^13^	*U*^23^
Br	0.0555 (4)	0.0544 (4)	0.0722 (5)	−0.0345 (3)	−0.0077 (3)	−0.0112 (3)
ClA	0.086 (2)	0.0646 (16)	0.118 (2)	−0.0415 (14)	0.0202 (16)	−0.0107 (14)
ClB	0.086 (2)	0.0646 (16)	0.118 (2)	−0.0415 (14)	0.0202 (16)	−0.0107 (14)
S	0.0433 (8)	0.0443 (8)	0.0292 (7)	−0.0198 (7)	−0.0030 (6)	−0.0056 (6)
O1	0.039 (2)	0.041 (2)	0.0288 (19)	−0.0193 (17)	−0.0074 (16)	−0.0062 (16)
O2	0.044 (3)	0.040 (3)	0.174 (6)	−0.012 (2)	−0.022 (3)	−0.040 (3)
O3	0.045 (3)	0.046 (3)	0.087 (3)	−0.014 (2)	−0.021 (2)	−0.006 (2)
O4	0.061 (3)	0.065 (3)	0.041 (2)	−0.022 (2)	−0.001 (2)	−0.027 (2)
C1	0.033 (3)	0.033 (3)	0.030 (3)	−0.013 (2)	−0.005 (2)	−0.009 (2)
C2	0.030 (3)	0.023 (2)	0.029 (3)	−0.005 (2)	−0.002 (2)	−0.009 (2)
C3	0.039 (3)	0.033 (3)	0.038 (3)	−0.012 (2)	−0.004 (2)	−0.014 (2)
C4	0.039 (3)	0.033 (3)	0.049 (3)	−0.017 (2)	−0.002 (3)	−0.015 (3)
C5	0.040 (3)	0.035 (3)	0.040 (3)	−0.015 (2)	0.001 (3)	−0.007 (2)
C6	0.042 (3)	0.039 (3)	0.030 (3)	−0.014 (3)	−0.004 (2)	−0.007 (2)
C7	0.029 (3)	0.028 (3)	0.039 (3)	−0.007 (2)	−0.006 (2)	−0.012 (2)
C8	0.033 (3)	0.031 (3)	0.033 (3)	−0.012 (2)	−0.001 (2)	−0.009 (2)
C9	0.036 (3)	0.045 (3)	0.037 (3)	−0.020 (3)	−0.005 (2)	−0.012 (3)
C10	0.045 (4)	0.041 (3)	0.048 (4)	−0.016 (3)	−0.003 (3)	−0.012 (3)
C11A	0.059 (4)	0.062 (4)	0.090 (5)	−0.019 (3)	−0.002 (4)	−0.032 (4)
C12A	0.170 (8)	0.120 (7)	0.171 (9)	−0.051 (6)	0.035 (7)	−0.037 (7)
C11B	0.059 (4)	0.062 (4)	0.090 (5)	−0.019 (3)	−0.002 (4)	−0.032 (4)
C12B	0.170 (8)	0.120 (7)	0.171 (9)	−0.051 (6)	0.035 (7)	−0.037 (7)
C13	0.055 (4)	0.051 (4)	0.057 (4)	−0.010 (3)	−0.017 (3)	−0.012 (3)

### Geometric parameters (Å, °)

**Table:**

Br—C4	1.907 (5)	C5—H5	0.9300
ClA—C12A	1.559 (19)	C6—C7	1.375 (7)
ClB—C12B	1.65 (3)	C6—H6	0.9300
S—O4	1.498 (4)	C8—C9	1.494 (7)
S—C1	1.766 (5)	C9—C10	1.505 (8)
S—C13	1.788 (6)	C9—H9A	0.9700
O1—C8	1.377 (6)	C9—H9B	0.9700
O1—C7	1.378 (6)	C11A—C12A	1.459 (15)
O2—C10	1.328 (7)	C11A—H11A	0.9700
O2—C11A	1.448 (9)	C11A—H11B	0.9700
O2—C11B	1.65 (2)	C12A—H12A	0.9700
O3—C10	1.190 (7)	C12A—H12B	0.9700
C1—C8	1.350 (7)	C11B—C12B	1.45 (2)
C1—C2	1.445 (7)	C11B—H11C	0.9700
C2—C3	1.395 (7)	C11B—H11D	0.9700
C2—C7	1.396 (7)	C12B—H12C	0.9700
C3—C4	1.380 (7)	C12B—H12D	0.9700
C3—H3	0.9300	C13—H13A	0.9600
C4—C5	1.385 (8)	C13—H13B	0.9600
C5—C6	1.383 (7)	C13—H13C	0.9600
			
O4—S—C1	105.9 (2)	C10—C9—H9B	109.2
O4—S—C13	106.1 (3)	H9A—C9—H9B	107.9
C1—S—C13	98.5 (3)	O3—C10—O2	123.5 (5)
C8—O1—C7	106.5 (4)	O3—C10—C9	126.4 (5)
C10—O2—C11A	116.1 (6)	O2—C10—C9	110.0 (5)
C10—O2—C11B	109.4 (13)	O2—C11A—C12A	108.9 (13)
C11A—O2—C11B	31.3 (12)	O2—C11A—H11A	109.9
C8—C1—C2	107.4 (4)	C12A—C11A—H11A	109.9
C8—C1—S	123.8 (4)	O2—C11A—H11B	109.9
C2—C1—S	128.7 (4)	C12A—C11A—H11B	109.9
C3—C2—C7	119.1 (5)	H11A—C11A—H11B	108.3
C3—C2—C1	136.0 (5)	C11A—C12A—ClA	128.4 (16)
C7—C2—C1	104.9 (4)	C11A—C12A—H12A	105.2
C4—C3—C2	116.8 (5)	ClA—C12A—H12A	105.2
C4—C3—H3	121.6	C11A—C12A—H12B	105.2
C2—C3—H3	121.6	ClA—C12A—H12B	105.2
C3—C4—C5	123.3 (5)	H12A—C12A—H12B	105.9
C3—C4—Br	118.2 (4)	C12B—C11B—O2	106 (4)
C5—C4—Br	118.4 (4)	C12B—C11B—H11C	110.5
C6—C5—C4	120.3 (5)	O2—C11B—H11C	110.5
C6—C5—H5	119.9	C12B—C11B—H11D	110.5
C4—C5—H5	119.9	O2—C11B—H11D	110.5
C7—C6—C5	116.6 (5)	H11C—C11B—H11D	108.7
C7—C6—H6	121.7	C11B—C12B—ClB	110 (3)
C5—C6—H6	121.7	C11B—C12B—H12C	109.7
C6—C7—O1	126.0 (5)	ClB—C12B—H12C	109.7
C6—C7—C2	123.8 (5)	C11B—C12B—H12D	109.7
O1—C7—C2	110.3 (4)	ClB—C12B—H12D	109.7
C1—C8—O1	110.9 (4)	H12C—C12B—H12D	108.2
C1—C8—C9	133.1 (5)	S—C13—H13A	109.5
O1—C8—C9	115.9 (4)	S—C13—H13B	109.5
C8—C9—C10	112.0 (4)	H13A—C13—H13B	109.5
C8—C9—H9A	109.2	S—C13—H13C	109.5
C10—C9—H9A	109.2	H13A—C13—H13C	109.5
C8—C9—H9B	109.2	H13B—C13—H13C	109.5
			
O4—S—C1—C8	−135.7 (5)	C1—C2—C7—O1	−1.1 (5)
C13—S—C1—C8	114.8 (5)	C2—C1—C8—O1	−0.5 (6)
O4—S—C1—C2	39.3 (5)	S—C1—C8—O1	175.4 (3)
C13—S—C1—C2	−70.2 (5)	C2—C1—C8—C9	176.0 (5)
C8—C1—C2—C3	−178.3 (6)	S—C1—C8—C9	−8.1 (9)
S—C1—C2—C3	6.0 (9)	C7—O1—C8—C1	−0.1 (5)
C8—C1—C2—C7	0.9 (5)	C7—O1—C8—C9	−177.3 (4)
S—C1—C2—C7	−174.7 (4)	C1—C8—C9—C10	−71.8 (8)
C7—C2—C3—C4	1.7 (7)	O1—C8—C9—C10	104.6 (5)
C1—C2—C3—C4	−179.1 (5)	C11A—O2—C10—O3	−5.9 (11)
C2—C3—C4—C5	0.7 (8)	C11B—O2—C10—O3	27.3 (16)
C2—C3—C4—Br	179.5 (4)	C11A—O2—C10—C9	175.0 (7)
C3—C4—C5—C6	−2.4 (8)	C11B—O2—C10—C9	−151.7 (14)
Br—C4—C5—C6	178.8 (4)	C8—C9—C10—O3	21.9 (9)
C4—C5—C6—C7	1.5 (8)	C8—C9—C10—O2	−159.1 (5)
C5—C6—C7—O1	179.8 (5)	C10—O2—C11A—C12A	162.3 (11)
C5—C6—C7—C2	0.9 (8)	C11B—O2—C11A—C12A	78 (3)
C8—O1—C7—C6	−178.2 (5)	O2—C11A—C12A—ClA	−36 (3)
C8—O1—C7—C2	0.8 (5)	C10—O2—C11B—C12B	176 (3)
C3—C2—C7—C6	−2.6 (8)	C11A—O2—C11B—C12B	−76 (4)
C1—C2—C7—C6	178.0 (5)	O2—C11B—C12B—ClB	52 (6)
C3—C2—C7—O1	178.4 (4)		

### Hydrogen-bond geometry (Å, °)

**Table:**

*D*—H···*A*	*D*—H	H···*A*	*D*···*A*	*D*—H···*A*
C11A—H11B···Cg^i^	0.97	3.07	3.779 (7)	131
C3—H3···O4^ii^	0.93	2.60	3.483 (7)	159
C5—H5···O3^iii^	0.93	2.58	3.423 (7)	150
C9—H9A···O1^iv^	0.97	2.60	3.545 (6)	165
C9—H9B···O4^v^	0.97	2.34	3.294 (7)	170

Symmetry codes: (i) *x*, *y*−1, *z*; (ii) −*x*+1, −*y*+1, −*z*+1; (iii) −*x*+1, −*y*+1, −*z*; (iv) −*x*, −*y*+1, −*z*; (v) −*x*, −*y*+1, −*z*+1.
